# Between-Session Reliability of Field-Based Assessments of Isometric Neck Strength

**DOI:** 10.3390/s24155015

**Published:** 2024-08-02

**Authors:** Samuel W. Oxford, Neil D. Clarke, Jason Tallis

**Affiliations:** 1 Centre for Physical Activity Sport and Exercise Science, Coventry University, Priory Street, Coventry CV15FB, UK; ab0289@coventry.ac.uk; 2 College of Life Sciences, Faculty of Health, Education and Life Sciences, Birmingham City University, Edgbaston, Birmingham B153TN, UK; neil.clarke@bcu.ac.uk

**Keywords:** reliability, isometric neck strength, concussion, force–time curve, time-specific force, validity

## Abstract

The reliability of the fixed-frame dynamometer for measuring isometric neck strength is established, but with limited field-based applications. This study aimed to establish the inter- and intra-session reliability of the peak force for neck flexors, extensors, and side flexors using the VALD ForceFrame and DynaMo and the force–time characteristics in the quadruped position (ForceFrame). Twenty-seven recreationally active males performed three repetitions of isometric neck flexion, extension, and side flexion over two sessions in random order using the VALD ForceFrame and DynaMo. Both devices demonstrated acceptable reliability, with the Forceframe ICC > 0.8 and CV% < 13.8% and the DynaMo ICC > 0.76 and CV% < 13.8%. No systematic or proportional differences were found using the Passing–Bablock procedure, and Bland–Altman analysis confirmed agreement across measures. Reliability was shown for right-side (ICC > 0.76) and left-side (ICC > 0.79) flexion and flexion (ICC > 0.75) across 50, 100, 150, and 200 ms. Statistical parametric mapping indicated no differences in ForceFrame-generated isometric force–time curves between sessions, though the CV was highest in the force development phase. The findings suggest that both tools can reliably assess neck strength, supporting their use in sports and clinical settings. However, assessment methods are not interchangeable, emphasising the need for standardised neck strength assessment approaches.

## 1. Introduction

Sports-related concussion (SRC) [1] and repetitive subconcussive forces are of major concern across a multitude of sports, but especially contact sports. Sports that involve repetitive impacts to the head, neck, and body, whether intentional or unintentional, can cause movement of the brain within the skull [2,3]. Although subconcussive impacts do not present with clinical signs and symptoms, long-term exposure can result in structural and functional brain changes [3]. The importance of strong neck musculature in mitigating these structural changes has received significant attention over the last decade [4]. The importance of a strong neck has been shown to reduce the incidence of concussion within a season in male professional rugby union players [5]. However, there are several concerns about the lack of consistency with respect to the strength measurement techniques currently being used [6,7]. Considering the potential importance of neck muscle strength in reducing the risk of SRC and subconcussive impacts, there is no consensus on the most appropriate method for assessment. 

Neck strength has been assessed through maximal isometric contractions (MVICs) involving flexion, extension, and side flexion in various positions: seated [5,8,9,10], standing [11], lying down, and in a quadrupedal stance [12]. These assessments have all involved a make test, where a load cell is connected either to a fixed frame or through a tethered system, and participants push/pull against the load cell. The quadruped position has shown good inter- and intratester reliability [12]; however, the application of the COSMIN checklist and taxonomy identified the need for further research on the measurement properties, as not all items were evaluated; for example, it was not compared to another method. It is crucial for practitioners to use reliable and valid methods that ensure conclusions about the measurement properties of the instrument [13] when assessing individuals to inform practice. It is essential that normative data on neck strength can be used to develop an understanding of the relationship between neck strength, (1) head accelerations, and (2) SRC and thus help guide practitioners in return-to-play and rehabilitation protocols. Currently, measurements of neck strength are hampered by a lack of evidence and consensus on what the best method is [14,15] to assess neck strength, thus leading to non-comparable findings, a risk of incorrect conclusions, and non-evidence-based practices. Therefore, there is value in comparing a neck assessment protocol that has demonstrated good reliability, such as the quadruped position with the ForceFrame [12], with an alternative meter in the same position. This approach would provide an evidence-based rationale for endorsing the quadruped position as the recommended method, facilitating comparisons across sports and contributing to the establishment of baseline measures for neck strength.

The current evaluation of the strength of the neck musculature is specific to maximal voluntary peak isometric force in flexion, extension, and side flexion, with greater muscle strength being associated with decreased linear and angular velocities of the head during collisions [16]. However, the evaluation of the rapid-force-producing capability of the neck musculature may provide important insight for profiling and monitoring. Therefore, it would be prudent to inspect an athlete’s capacity to generate force at time-specific points as an indicator of the neck musculature’s ability to generate force rapidly. This assessment could provide evidence of the crucial role the neck musculature plays in stabilising the head during collisions, potentially reducing the forces transmitted to the brain and lowering the risk of sports-related concussion (SRC). 

Among practices from other well-established isometric tests, the isometric midthigh pull (IMTP) has demonstrated high reliability [17,18] and low measurement error when force is evaluated at different time epochs associated with the force development phase [19,20]. This study is the first to investigate the inter-session and intra-session reliability of the force–time characteristics of the neck musculature, building on previous work that assessed the retest reliability of isometric neck strength force–time characteristics [8]. Prior research demonstrated acceptable reliability, with ICC values ranging from 0.9 to 0.99 for peak force, for time to 50% of peak force and the average rate of force development (RFD). A limitation of this investigation was the use of a custom-made rig with the participants in a seated position, which makes it difficult to apply to clinical settings. While the results of the previous work are of value, time to peak force and the average rate of force development may not provide the best indication of rapid force generation, which, in the context of neck muscle activation, is important for further understanding head accelerations and the potential relationship of neck strength with SRC. Thus, using the assessment guidelines from the IMTP [21], a well-established test for evaluating lower limb maximal isometric strength, the present study evaluated force generation at several time epochs. 

Focusing on individual force–time variables can offer valuable information about neck strength characteristics. However, relying solely on isolated data points or specific test phases may hinder the identification of subtle performance changes that can be better understood by examining the entire force–time curve. For example, Hughes et al. [22] demonstrated that a curve analysis of vertical jump force–time profiles was more effective for detecting neuromuscular fatigue than discrete force–time variables. Such analysis may therefore be important in the evaluation of neck strength, where establishing the repeatability of the isometric force–time curve is an important first step for understanding the value in monitoring time-series data. 

Given the potential importance of the force–time characteristics of muscles of the neck in mitigating head acceleration and potentially reducing the risk of sports-related concussion (SRC), there is a requirement to establish reliable assessment methods. Therefore, this study set out to (1) determine the reliability of the neck assessment using the quadruped position with a tethered load cell (VALD DynaMo) and a fixed-frame dynamometer (VALD ForceFrame), (2) determine the effect of the test direction on peak isometric force for both assessment methods, and (3) determine the reliability of the force–time data of the VALD ForceFrame. 

## 2. Materials and Methods

This study used a double-session repeated-measures research design, whereby isometric neck strength was assessed in flexion, extension, and left- and right-side flexion with the VALD ForceFrame and VALD DynaMo (VALD Performance, Brisbane, Queensland, Australia) in the quadruped position in a randomised order. Three trials in each direction with both pieces of equipment were performed on two occasions separated by seven–eight days and scheduled at the same time of day to account for fatigue and any daily variations. 

A convenience sample of *n* = 27 male recreationally active university staff and/or students aged 28 ± 8 y with a mass of 78.7 ± 10.5 kg and a height of 175.8 ± 20.5 cm were recruited via word of mouth. A required sample size of *n* = 14 was determined without a potential 10% loss to follow-up, in accordance with the estimation approach [23] (expected reliability (ICC) (ρ): 0.8; precision (± expected) 0.20; Confidence Level 100(1 − α): 95%; number of repetitions (k): 2). Any participants reporting neck pain, cervical injury, or having experienced a concussion in the previous month were excluded from the study. Participants were informed about the study and gave written consent prior to participation. Ethics approval was provided by the University Human Research Ethics Committee (P144472). 

Measurements of height (to the nearest 0.5 cm) and body mass (to the nearest 0.5 kg) were recorded on the first visit to the laboratory. Following a standardised warm-up [12], each participant completed three repetitions, with 10 s between contractions, a 60 s rest between directions, and a five-minute break between setups. The order of testing was randomised with regard to equipment and the order of movements for each participant. The order was kept the same for the follow-up visit. 

**Figure 1:**
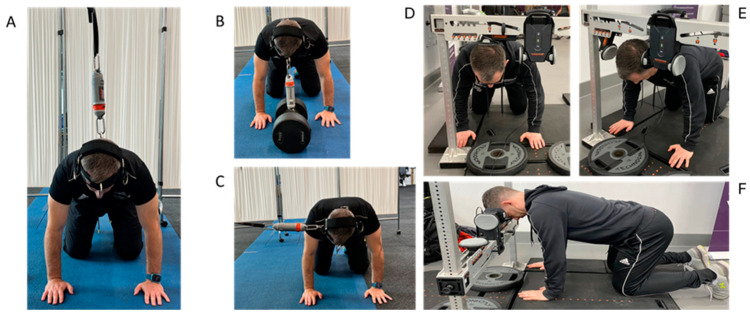
Test position adopted for (**A**) = flexion, (**B**) = extension, and (**C**) = side flexion for the VALD DynaMo and (**D**) = extension, (**E**) = side flexion, and (**F**) = flexion for the VALD ForceFrame for the assessment of isometric neck strength.

The protocol followed previously reported procedures [12]. Specifically, participants were instructed to assume the quadruped (start) position: hands shoulder-width apart perpendicularly below the proximal joint, scapulae drawn together, elbows fully extended, and hips and knees set at 90 degrees. A head harness (Alpha+, Iron Neck, Austin, TX, USA) was secured around the head in accordance with manufacturing guidelines, with the D-ring anchor points lined up with the frontal bone superior to the eyebrows for flexion; the occiput for extension; and the temporal bone just above the superior aspect of the ear helix for left- and right-side flexion in the same location as the load cell of the ForceFrame [12]. The portable handheld dynamometer VALD DynaMo was connected to the head harness via a carabiner and a non-stretch cord fixed to a metal frame (Figure 1) and recorded in VALD DynaMo v1.6.1 (VALD Performance, Brisbane, Queensland, Australia) via Bluetooth to an iPad device with a sampling frequency of 225 Hz. The VALD ForceFrame was hardwired to a PC (HP ZBOOK) and recorded in VALD ForceFrame v3.14 (VALD Performance, Brisbane, Queensland, Australia) at a sampling frequency of 400 Hz. 

Pre-test, participants became familiar with pushing against the load cell or harness at an estimated 80% MVIC. The participants were instructed to push as fast and as hard as possible for three seconds, and the peak MVIC was recorded for each of the isometric actions [12]. The tests were administered by the same experienced strength and conditioning coach, who was unaware of the scores obtained in the initial testing session.

The force–time curve assessment was conducted with the VALD ForceFrame only due to the inability to access raw data from the VALD DynaMo. All force–time data were inspected using a custom-made Excel spreadsheet to determine specific force–time characteristics. The maximum force generated throughout the contraction was reported as peak force (PF). In addition, time-specific force values at 50 ms (Force50), 100 ms (Force100), 150 ms (Force150), and 200 ms (Force200) were calculated. The onset threshold of the movement was set at 40 N across all four directions, which has previously demonstrated high reliability in the IMTP [21]. 

### Statistical Analysis

Descriptive statistics (mean ± SD) were calculated for peak force (N) in each of the four directions. Of the starting participants, 74% (n = 20) completed both sessions due to dropout; thus, their initial values were excluded from the reliability analysis. A one-way analysis of variance was used to compare the peak isometric neck force between the four directions for each session. Mauchly’s test of sphericity was used to determine whether sphericity was violated, and a Greenhouse–Geisser correction was conducted where applicable. Where differences were noted in ANOVA, Bonferroni-adjusted post hoc tests were used to identify where significant differences occurred. The effect size for the ANOVA statistics was estimated using partial Eta-squared (η^2^_p_).

To determine the relative reliability of the measures, intraclass correlation coefficients (ICC_(3,1)_) were calculated for the peak force values from the three trials for each of the four directions [24]. The CV was calculated based on the mean square error term of logarithmically transformed data [25]. The ICCs were evaluated using the following criterion measures: values less than 0.5 indicate poor reliability, values between 0.5 and 0.75 indicate moderate reliability, values between 0.75 and 0.9 indicate good reliability, and values greater than 0.90 indicate excellent reliability. Acceptable reliability was defined as an ICC_(3,1)_ > 0.70 and a CV of <15% [26]. The absolute reliability of the peak isometric force was determined using the standard error of measurement (SE_m_) calculated using the formula SE_m_ *=* SD × √ (1 − ICC), where the SD value was the combined SD value from the two trials, and the ICC values were the two-way mixed-model single measure of consistency. The minimal detectable change (MDC) was determined using the formula MDC = 1.96 × √2 × SE_m_ [27].

The peak values recorded in the three trials for each test direction were utilised to compare the VALD DynaMo and the ForceFrame. To evaluate the distribution, flexion, extension, and left- and right-side flexion, scatterplots with regression confidence were created. The normal distribution of the variables was assessed using Shapiro–Wilk’s tests. A two-tailed dependent *t*-test was employed to determine the differences in values between the two methods. The magnitude of the difference was quantified using Cohen’s *d* effect size and was considered trivial when *d* < 0.2, small when *d* < 0.5, moderate when *d* < 0.8, and large when *d* > 0.8 [28]. Bland–Altman plots were generated to evaluate the bias and variability between the two methods in each of the four directions, with 95% limits of agreement (LOA) defined as the mean difference ± 1.96 standard deviation [29]. 

Systematic and proportional disparities between the DynaMo and the ForceFrame were assessed with the Passing–Bablock regression equation as previously described [30]. The findings were interpreted as follows: if 0 is in the 95% CI of “a”, and 1 is in the 95% CI of “b”, the two methods are comparable. If 0 is not in the 95% CI of ‘‘a’’, there is a systematic difference, and if 1 is not in the 95% CI of “b”, then there is a proportional difference between the two methods [30]. Statistical analysis was performed using IBM SPSS Statistics version 26, and the criterion for statistical significance was set at *p* ≤ 0.05.

Differences across the force–time curve were assessed using statistical parametric mapping (SPM) using the SPM–1D package (Todd Pataky, v.M0.1; available at “http://www.spm1d.org/ (accessed on 9 April 2024)”, via MATLAB (The MathWorks Inc., R2021a, Natick, MA, USA) [31]. Initially, data were cropped to 750 data points to prevent variations in the descending arm of the force–time curve from affecting the analysis. Data were then temporally normalised using linear interpolation to 101 data points. For each assessment, a two-sample SPM[t] (two-sided *t*-test) was conducted to assess the between-session differences in the performance of the repetition that elicited the highest force. For the data obtained in the second visit, differences between repetitions were analysed using one-way repeated-measures ANOVA (SPM F) with post hoc paired *t*-tests (SPM t) as per previous work [32]. SPM calculates the t or F statistic for every data point, but instead of calculating a *p*-value for every data point, inferential statistics are based on random field theory and thus maintain a constant error of α [33]. Where clusters crossed the critical threshold, this indicated a significant difference at *p* ≤ 0.05. In addition, using time-normalised data, individual coefficients of variation (CVs) were determined at each time point and averaged across the sample to provide further insight with respect to the between- and within-session reliability of the force–time curves. 

## 3. Results

Descriptive statistics for the three repetitions in each of the two trials in all four directions for both sets of equipment are presented in Table 1. The absolute and relative reliability values are reported in Table 1 for both trials. The inter-session reliability for the ForceFrame was excellent for both sessions (Table 1) in all four directions and ranged in value from 0.83 to 0.98 (5.8–9.9%), and that for the DynaMo was good to excellent, with values ranging from 0.7 to 0.98 (2.9–11.8%). 

Isometric neck strength with the DynaMo showed that there was a significant difference between directions for session 1 (*p* < 0.01, η^2^_p_ 0.22) (Table 1). Bonferroni post hoc analysis revealed a significant difference between flexion and both left-side flexion (30%) and right-side flexion (32%) (*p* < 0.05) for session 1 (Table 1), and for session 2, there was also a significant difference between directions (*p* < 0.01, Ƞ^2^_p_ 0.24). Bonferroni post hoc analysis revealed a significant difference between flexion and both left-side flexion (33%) and right-side flexion (34%) (*p* < 0.01). 

For the ForceFrame, there was a significant difference between directions in session 1 (*p* < 0.05, η^2^_p_ 0.46) (Table 1). Bonferroni post hoc analysis revealed a 25% significant difference between flexion and extension (*p* = 0.003) and between flexion and both left- (25%) and right-side flexion (32%) (*p* < 0.05) for session 1 (Table 1). There was a significant difference between extension and both left- (57%) and right-side flexion (65%) (*p* < 0.01), and for session 2, there was also a significant difference between directions (*p* < 0.01, Ƞ^2^_p_ 0.44). Post hoc analysis showed that there was a significant difference between flexion and both left- (29%) and right-side flexion (42%) (*p* < 0.01) and between extension and both left- (53%) and right-side flexion (69%) (*p* < 0.01) (Table 1). For extension and flexion, there was a non-significant difference of 19% (*p* = 0.51) (Table 1). 

Intra-rater reliability results from session 1 and session 2 from the single-measure ICCs were good to excellent across all directions, with an ICC > 0.8 and a CV% < 14% for the ForceFrame and an ICC > 0.76 and a CV% < 14% for the DynaMo (Table 1). The highest SE_m_ for the ForceFrame was achieved during Ext (21 N) for the group, indicating the highest level of variability in the four directions measured, whereas for the DynaMo, LSF (43 N) showed the highest level of variability (Table 1). When the MDC was compared with the overall mean for each direction, the following values were calculated to indicate whether a meaningful change for clinical practice had occurred in neck strength: for the ForceFrame: extension: 57 N; flexion: 50 N, left: 54 N; and right-side flexion: 51 N; for DynaMo: extension: 65 N; flexion: 50 N, left: 119 N; and right-side flexion: 63 N (Table 1).

**Figure 2:**
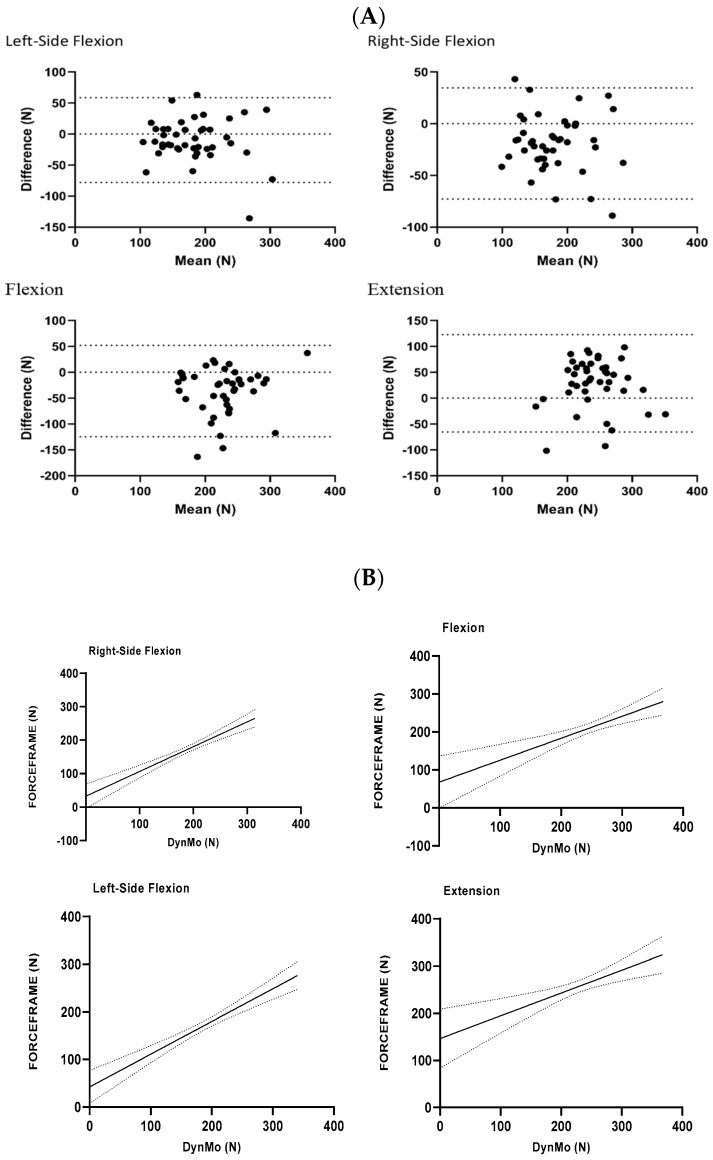
(**A**) Bland–Altman plots comparing the mean isometric neck strength between the VALD DynaMo and the VALD ForceFrame for left-side flexion, right-side flexion, and extension. The upper and lower lines represent the 95% limits of agreement between the two methods (mean ± 1.96 standard deviation). (**B**) Flexion, extension, and left- and right-side flexion scatterplots with regression confidence intervals between VALD ForceFrame and VALD DynaMo.

Overall, the mean for the DynaMo was significantly greater in flexion and right-side flexion and significantly lower in EXT (Table 2, *p* ≤ 0.05), with a −0.59 effect size. Scatterplots with linear regression confidence showed precision and linearity in all directions (Figure 2B). Absolute agreement between the values obtained with the DynaMo and the ForceFrame are shown in the Bland–Altman plots (Figure 2A). The mean bias ranged from −37 (N)- to −10 (N) for flexion and right- and left-side flexion and 28 (N) for extension. The Passing–Bablock regression analysis showed a good correlation between the two methods. Slopes ranged from 0.9 to 1.1 and intercepts from −64.5 to 29.4 across all directions, with no proportional or systematic difference in any of the directions tested with the DynaMo and ForceFrame (Table 3).

The inter-session reliability of the force–time curve data across all time points and directions showed inconsistencies in ICC values and CV values (Supplement Table S1). Acceptable ICCs for all force–time values were shown across flexion (ICCs, 0.75–0.78), left-side flexion (ICCs, 0.79–0.87), and right-side flexion (ICCs, 0.76–0.88) but not for extension (ICCs, 0.58–0.7) (Supplement Table S1). 

**Figure 3:**
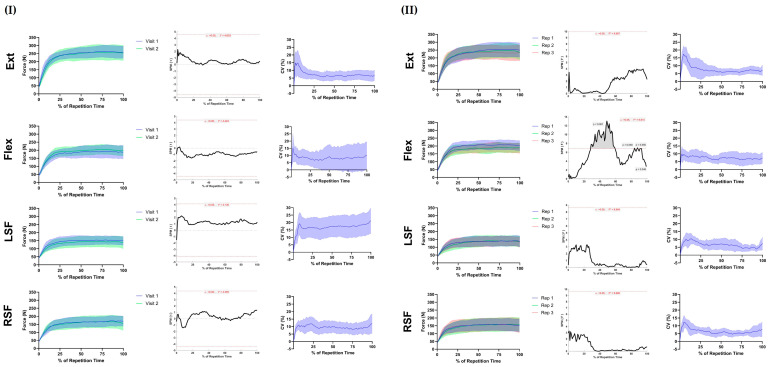
Comparison of between (**I**) and within-session (**II**) isometric neck strength force–time curves [left-hand panel = mean force (N) ± 95% CI; middle panel = results of SPM *t*-test; right-hand panel = mean CV (%) ± 95% CI; Ext = extension; Flex = flexion; LSF = left-side flexion; RSF = right-side flexion. N = 14 for Ext, Flex, and RSF; N = 13 for LSF].

Across all assessments, the results of SPM indicated that there was no difference in the force-time curve of the repetition that elicited the highest peak force at visit one compared to visit two (Figure 3I; *p* > 0.05 in all cases). The CV, when evaluated across the force–time curve, was assessment-specific (Figure 3I). For Ext and Flex, the mean CV increased to a peak within the first 10% of the assessment and then decreased and plateaued below 10% thereafter. For left-side flexion, the mean CV also increased in the first 10% of the trial and was sustained above 15% thereafter. Right-side flexion followed a similar pattern, peaking and plateauing at ~10%.

For extension and left- and right-side flexion, the results of SPM indicated that there were no differences in the force–time curve between the three consecutive repetitions (Figure 3I. *p* > 0.05 in all cases). However, for flexion, SPM ANOVA indicated a main effect of repetition between 28 and 59% (Figure 3II, *p* < 0.001), at 84% and 88% (Figure 3II, *p* = 0.05 in both cases), and between 92 and 93% (Figure 3II, *p* = 0.048) of the trial. The SPM paired *t*-test indicated that force between 49 and 54% (Supplement Figure S1B, *p* < 0.001) and at 56% (Supplement Figure S1B. *p* = 0.031) was higher in repetition 1 compared to repetition 3. The CV, when evaluated across the force–time curve, showed a similar pattern across tests. The mean CV typically peaked within the first 10% of the trial and decreased to a mean level below 10% thereafter.

## 4. Discussion

This is the first study to have established the inter- and intra-session reliability of neck strength measures in the quadruped position with the VALD ForceFrame and DynaMo. Both modes of measurement demonstrated acceptable to excellent inter-session reliability in all four directions (Table 1). For the VALD ForceFrame method, the interclass correlation coefficients (ICCs) were >0.83, and the coefficients of variation (CVs) were <11.8% (Table 1). Similarly, the DynaMo method showed ICC values > 0.7 and CVs < 11.8% (Table 1). Notably, both the ForceFrame and DynaMo inter-session ICC values surpassed the minimal acceptable thresholds of ICC > 0.7 and CV < 15% previously reported for isometric contractions in the midthigh pull exercise. 

The measurement of peak isometric force with the VALD DynaMo and the VALD ForceFrame showed that peak force was significantly greater in flexion (*p* < 0.05) compared to left- and right-side flexion, which is consistent with the previous finding of neck strength in the quadruped position [12]. However, there was no significant difference (*p >* 0.05, Table 1) between extension and flexion with the VALD DynaMo, which is contrary to the finding with the VALD ForceFrame, which showed a significant difference (*p <* 0.05) between flexion and extension. The peak force values of the ForceFrame are in line with those previously reported for the quadruped position [12], providing further evidence to support the use of the quadruped position in the assessment of isometric neck strength. However, further research is required to understand the impact that the test position has on the strength properties of the neck before a gold-standard methodology can be established. The potential reason for the differences between modes of assessment could be that the setup for the VALD DynaMo allowed for potential lateral, fore-and-aft, and rotational movements, as the head was supported in a head harness and not fixed as it is in the VALD ForceFrame. The greater movement could have allowed for compensatory movements from the upper and lower quadrants, allowing greater co-contraction and muscle recruitment, therefore resulting in greater MVICs. However, this is only conjecture, and further research is needed to understand the influence that the start position and equipment have on muscle recruitment and force production. 

The regression analysis of PF for both the VALD ForceFrame and DynaMo modes of measurement demonstrated excellent precision and linearity (Figure 2), with no systematic or proportional differences observed in any of the test directions (Table 2). Bland–Altman representation (Figure 2) further supported the agreement between the two methods for all directions, although there was a positive offset indicating that the VALD DynaMo test produced significantly (*p* < 0.001) higher strength values than the VALD ForceFrame in flexion and left- and right-side flexion (Table 2). Conversely, there was a negative offset in extension (Table 3), with moderate effect sizes across extension and left- and right-side flexion and a large effect size in flexion (Table 3). The mean bias detected for left- and right-side flexion and flexion ranged from −10 N to −37 N, while for extension, it was 28 N. This mean bias is notably lower than the bias reported in the comparison of a handheld dynamometer with a fixed dynamometer for the assessment of neck strength (1.8 kg to 3.8 kg) [11]. These findings indicate that the DynaMo does not provide the same values as the ForceFrame method and, therefore, cannot be used interchangeably for the assessment of isometric PF of the neck musculature in flexion, extension, and side flexion. 

Although the VALD DynaMo was shown not to be comparable to the VALD ForceFrame, it is a worthwhile process to determine the reliability of the equipment. The DynaMo may still be a useful tool for determining isometric neck strength in practical situations where it is not possible to have a fixed-frame dynamometer, such as in the field. Both the VALD ForceFrame and VALD DynaMo exhibited acceptable inter-session reliability, with ICC values > 0.8 and CVs < 13.8% for the VALD ForceFrame and ICC values > 0.84 and CVs < 13.8% for the VALD DynaMo (Table 1). These results are similar to previously published ICCs for intra-rater reliability for custom-made fixed-frame dynamometers (0.90–0.97) [34] and for other commercially available fixed-frame dynamometers: 0.96–0.99 [35] and 0.85–0.97 [36]. The findings of the current study show similar reliability results for the VALD ForceFrame [12] (ICCs ranging from 0.83 to 0.94 and CVs ranging from 5.2% to 14%) for the assessment of the neck musculature in the quadruped position; however, this is the first study to report the reliability findings in accordance with the COSMIN checklist for reliability [13]. This study therefore provides further evidence to support the recommendation that the quadruped position and the fixed-frame dynamometer be used as a standardised protocol for the assessment of isometric neck strength in flexion, extension, and left- and right-side flexion in future research and clinical practices. The current study only assessed reliability over two sessions with a maximum of eight days between sessions. Therefore, it would be prudent to further the current knowledge by investigating the effects of extraneous variables, such as the training load, fatigue, and injury, on the repeatability of measuring neck strength over a longer period. 

This study advances the research on the isometric PF of the neck musculature in flexion, extension, and side flexion, as it is the first study to report intra-session reliability (Supplement Table S1) for time to PF across the four directions. The findings show that time to PF is inconsistent between repetitions across all four directions (Supplement Table S1). The poor reliability of time to PF has also been previously reported within the MTIP literature [37]. This is an important finding, as time to PF is a well-reported metric within software packages and should therefore not be used as an indicator of performance. This is the first study, to the authors’ knowledge, that has reported time-specific force–time values across a range of epochs for the isometric neck strength values for extension, flexion, and left- and right-side flexion. The results indicate that there is poor within-session reliability across all time-specific force values (Supplement Table S1); however, when the mean values for the session repetitions are considered (Table 3), relative and absolute reliability is demonstrated for all epochs for left- and right-side flexion, with ICCs > 0.79 and CVs < 14.6% (Table 3). For flexion, acceptable relative reliability was demonstrated across all epochs (Supplement Table S1), but absolute reliability was only demonstrated at 50 and 100 ms (Table 3). Extension showed poor relative and absolute reliability across all epochs except for 150 ms, where only acceptable relative reliability was demonstrated (Table 3). The ability to generate force in short time intervals has been shown to be more important than the production of maximum force for many sports and dynamic tasks [38,39]. Therefore, this research presents a starting point for further research into the relationship between head accelerations and the ability to generate force at different time force–time epochs and demonstrates the need to explore reliability functions prior to usage, which is a limitation of much of the literature on neck strength assessment. 

Previous work indicates that the evaluation of the entire force–time trace may offer important additional insight compared to discrete force–time metrics alone [22]. However, establishing the repeatability of the force trace is an important first step in understanding the value of the approach for monitoring neck strength. The within-session reliability of the force–time curves typically follows the trends seen in the discrete data. Importantly, there were no between-session differences in the force–time curves among the assessed directions. Whilst the variation was typically greater during the force development phase, for extension, flexion, and right-side flexion, the CVs across the curves were acceptable. Interestingly, the left-side-flexion trace was less repeatable, which may be attributable to participants being right-side-dominant and having better body control when pushing to the right. With the exception of left-side flexion, these data indicate that the force–time curve may be acceptable for monitoring; however, much like the discrete variables, work is now needed to understand the ecological validity of such neck strength variables. 

## 5. Conclusions

The findings of this study demonstrate that both the VALD DynaMo and the VALD ForceFrame produce reliable inter- and intra-session PF values in healthy male participants for isometric neck strength in flexion, extension, and left- and right-side flexion in the quadruped position, but they cannot be used interchangeably due to a lack of agreement between them. In the quadruped position, the isometric peak force measured for flexion, extension, and side flexion is different depending on whether the VALD DynaMo or ForceFrame is used. Considering the current drive to better understand the impact of head injuries in sports and the links between a strong neck and the mitigation of these injuries, this study provides practitioners with guidance on examining the force–time characteristics and not just PF values for determining the function of the neck musculature with respect to head injuries. Sports medicine practitioners need to be careful when considering the methods used to quantify the strength profiles of the neck musculature of patients, as the methods and data analysis directly impact the reliability of the measurement. 

## Figures and Tables

**Table 1:** Inter- and intra-session reliability of peak force for session 1 and session 2 for the VALD DynaMo and the ForceFrame.

			Dynamo				ForceFrame			
			Direction							
		Variable	EXT	FLEX	LSF	RSF	EXT	FLEX	LSF	RSF
Intra Session (Session 1)	Rep 1	Peak F (N)	216 ± 36	241 ± 44	180 ± 54	178 ± 50	248 ± 52	198 ± 51	159 ± 51	161 ± 46
	Rep 2	Peak F (N)	215 ± 41	246 ± 46	183 ± 52	182 ± 51	241 ± 53	194 ± 55	161 ± 52	162 ± 45
	Rep 3	Peak F (N)	221 ± 49	246 ± 42	186 ± 54	182 ± 50	241 ± 49	186 ± 60	156 ± 51	165 ± 51
	Rep 1–2	ICC	0.86	0.96	0.94	0.95	0.94	0.93	0.98	0.95
	Rep 1–2	95% CI	0.72–0.94	0.91–0.98	0.87–0.97	0.89–0.98	0.86–0.97	0.86–0.97	0.96–0.99	0.9–0.98
	Rep 1–2	SEm	14	9	13	11	13	14	7	10
	Rep 1–2	MDC	40	25	36	31	35	38	20	28
	Rep 1–2	CV %	7.2	4.1	7.5	6.9	6.8	7.8	4.6	7.1
	Rep 1–2	CV 95% CI	5.6–10.1	3.2–5.7	5.8–10.5	5.3–9.6	5.3–9.5	6.1–10.9	3.6–6.4	5.6–9.9
	Rep 2–3	ICC	0.83	0.96	0.97	0.93	0.96	0.87	0.97	0.95
	Rep 2–3	95% CI	0.66–0.92	0.92–0.98	0.93–0.99	0.86–0.97	0.91–0.97	0.74–0.94	0.93 –0.99	0.90–0.98
	Rep 2–3	SEm	18	9	9	13	10	57	51	11
	Rep 2–3	MDC	51	24	25	37	28	158	141	29
	Rep 2–3	CV %	8.9	3.8	5.1	7.9	5.5	11.6	5.9	7.1
	Rep 2–3	CV 95% CI	6.9–12.5	3.0–5.3	4–7.1	6.2–11.1	4.3–7.6	9.0–16.2	4.6 –8.2	5.6–9.9
	Rep 1–3	ICC	0.76	0.87	0.93	0.8	0.88	0.86	0.92	0.9
	Rep 1–3	95% CI	0.59–0.88	0.76–0.94	0.69–0.92	0.65–0.90	0.78–0.94	0.74–0.93	0.84 –0.96	0.81–0.95
	Rep 1–3	SEm	21	15	14	22	17	21	14	15
	Rep 1–3	MDC	58	43	40	61	48	57	40	42
	Rep 1–3	CV %	8.7	5.3	9.2	10.8	6.6	9.9	7.7	8
	Rep 1–3	CV 95% CI	6.7–12.2	4.1–7.3	7.1–12.9	8.4–15.2	5.2–9.2	7.7–13.8	6.0–10.8	6.2–11.1
Intra Session (Session 2)	Rep 1	Peak F (N)	207 ± 38	233 ± 39	170 ± 46	172 ±49	244 ± 46	208 ± 42	141 ± 39	158 ± 52
	Rep 2	Peak F (N)	208 ± 53	235 ± 42	173 ± 49	173 ± 51	237 ± 46	194 ± 42	138 ± 45	153 ± 47
	Rep 3	Peak F (N)	206 ± 50	238 ± 47	172 ± 50	173 ± 52	233 ± 45	193 ± 40	137 ± 43	153 ± 46
	Rep 1–2	ICC	0.78	0.98	0.97	0.96	0.94	0.9	0.94	0.95
	Rep 1–2	95% CI	0.52–0.91	0.92–0.99	0.92–0.99	0.89–0.98	0.84–0.97	0.76–0.96	0.85–0.97	0.88–0.98
	Rep 1–2	SEm	21	10	8	10	10	13	10	11
	Rep 1–2	MDC	59	27	23	29	27	35	27	30
	Rep 1–2	CV %	11.8	2.9	5.5	6.8	5.8	7.5	8.6	7.6
	Rep 1–2	CV 95% CI	8.7–18.1	2.2–4.4	4.1–8.1	5.1–10.1	4.4–8.5	5.6–11.1	6.5–12.8	5.8–11.4
	Rep 2–3	ICC	0.91	0.94	0.97	0.97	0.93	0.95	0.97	0.95
	Rep 2–3	95% CI	0.77–0.96	0.85–0.98	0.92–0.99	0.91–0.99	0.84–0.97	0.87–0.98	0.92–0.99	0.88–0.98
	Rep 2–3	SEm	15	11	9	9	11	37	43	10
	Rep 2–3	MDC	42	30	24	26	29	103	118	28
	Rep 2–3	CV %	7.9	5.2	5.7	6	6.2	5.5	6.5	7.4
	Rep 2–3	CV 95% CI	5.9–12.1	3.9–8.0	4.3–8.4	4.5–8.9	4.7–9.2	4.2–8.2	4.9–9.6	5.6–10.9
	Rep 1–3	ICC	0.69	0.87	0.92	0.91	0.83	0.91	0.89	0.93
	Rep 1–3	95% CI	0.47–0.86	0.72–0.95	0.81–0.96	0.80–0.96	0.66–0.92	0.81–0.96	0.76–0.95	0.83–0.97
	Rep 1–3	SEm	24	15	14	16	16	12	13	13
	Rep 1–3	MDC	68	43	38	44	45	32	37	36
	Rep 1–3	CV %	10.8	6.2	6.6	7	7.2	5.2	8.5	6.4
	Rep 1–3	CV 95% CI	8.0–16.6	4.6–9.4	5.0–9.8	5.3–10.5	5.4–10.7	3.9–7.7	6.4–12.7	4.8–9.4
Inter Session	Session 1	Peak F (N)	226 ± 49	248 ± 47 ^a^	190 ± 47 ^a^	187 ± 53 ^a^	263 ± 44 ^b^	210 ± 41 ^bc^	170 ± 49 ^bc^	160 ± 50 ^bc^
	Session 2	Peak F (N)	217 ± 48	241 ± 43 ^a^	181 ± 51 ^a^	179 ± 48 ^a^	250 ± 48 ^b^	210 ± 42 ^bc^	163 ± 9.4 ^bc^	148 ± 45 ^bc^
	Session 1–2	ICC	0.76	0.84	0.78	0.8	0.8	0.83	0.84	0.85
	Session 1–2	95% CI	0.48–0.90	0.65–0.94	0.53–0.91	0.55–0.91	0.57–0.92	0.62–0.93	0.64–0.93	0.65–0.94
	Session 1–2	SEm	24	18	43	23	21	17	19	18
	Session 1–2	MDC	65	50	119	63	57	47	54	51
	Session 1–2	CV %	10.9	8.3	13.8	13.6	9.7	9.4	13.2	13.8
	Session 1–2	CV 95% CI	8.1–16.2	6.2–12.3	10.3–20.8	10.2–20.4	7.3–14.4	7.0–14	9.9–19.9	10.3–20.8

ICC = intraclass correlation coefficient; 95% confidence interval for ICC_(3,1)_ single measure; SE_m_ = standard error of measurement; MDC = minimal detectable change; EXT = extension; FLEX = flexion; LSF = left-side flexion; RSF = right-side flexion. ^a^ Significant difference between FLEX and LSF and RSF in sessions 1 and 2 for DynaMo, *p* ≤ 0.05. ^b^ Significant difference between EXT and FLEX, LSF, and RSF in sessions 1 and 2 for ForceFrame, *p* ≤ 0.05. ^c^ Significant difference between FLEX and LSF and RSF in sessions 1 and 2 for ForceFrame, *p* ≤ 0.05.

**Table 2:** Inter-session reliability of the isometric neck strength force–time characteristics for the ForceFrame.

				ICC				CV	
	Variable	Session 1	Session 2	ICC	95% CI	SEm	MDC	%	95% CI
Extension	Time to peak force (s)	1.4 ± 0.7	1.4 ± 0.5	0.63	0.18–0.87	0.36	1.66	34.7	24.1–61.6
	Force at 50 ms (N)	94 ± 30	80 ± 25	0.58	0.10–0.84	18.20	11.82	22.7	16.0–39.0
	Force at 100 ms (N)	138 ± 47	121 ± 41	0.64	0.19–0.87	26.47	14.26	24.7	17.3–42.6
	Force at 150 ms (N)	169 ± 51	156 ± 54	0.70	0.30–0.89	28.62	14.83	21.8	15.4–37.4
	Force at 200 ms (N)	190 ± 53	180 ± 61	0.65	0.20–0.87	33.16	15.96	22.7	16.0–39.0
Flexion	Time to peak force (s)	1.5 ± 0.7	1.5 ± 0.5	0.60	0.13–0.85	0.39	1.72	36.2	25.1–64.4
	Force at 50 ms (N)	67 ± 15	70 ± 14	0.78	0.45–0.92	6.68	7.16	10.7	7.6–17.7
	Force at 100 ms (N)	96 ± 26	101 ± 25	0.75	0.37–0.91	12.48	9.79	15.5	11.0–26.1
	Force at 150 ms (N)	116 ± 34	126 ± 33	0.78	0.44–0.92	24.51	13.72	16.1	11.4–27.2
	Force at 200 ms (N)	129 ± 39	142 ± 37	0.78	0.44–0.92	17.78	11.69	16.1	11.4–27.2
LSF	Time to peak force (s)	1.7 ± 0.6	1.5 ± 0.5	0.66	0.22–0.88	0.34	1.62	27.2	19.1–47.4
	Force at 50 ms (N)	65 ± 12	60 ± 12	0.83	0.54–0.94	5.02	6.21	8.6	6.2–14.2
	Force at 100 ms (N)	87 ± 15	79 ± 25	0.79	0.46–0.93	11.39	9.35	14.4	10.2–24.2
	Force at 150 ms (N)	104 ± 36	94 ± 35	0.84	0.58–0.95	14.06	10.39	14.6	10.4–24.5
	Force at 200 ms (N)	113 ± 41	104 ± 42	0.87	0.65–0.96	14.66	10.61	13.9	9.9–23.3
RSF	Time to peak force (s)	1.6 ± 0.6	1.5 ± 0.6	0.45	−0.08–0.78	0.44	1.85	44.8	30.8–81.5
	Force at 50 ms (N)	67 ± 11	67 ± 11	0.78	0.43–0.92	5.14	6.29	8.7	6.3–14.5
	Force at 100 ms (N)	88 ± 20	91 ± 23	0.76	0.40–0.92	10.42	8.95	13.7	9.7–22.9
	Force at 150 ms (N)	102 ± 28	109 ± 33	0.85	0.59–0.95	11.83	9.54	13.3	9.5–22.2
	Force at 200 ms (N)	115 ± 33	121 ± 40	0.88	0.68–0.96	12.43	9.77	12.1	8.6–20.2

ICC = intraclass correlation coefficient; 95% confidence interval for the ICC_(3,1)_ single measure; SE_m_ = standard error of measurement; MDC = minimal detectable change; LSF = left-side flexion; RSF = right-side flexion.

**Table 3:** Comparison of the VALD DynaMo vs. ForceFrame tests using Bland–Altman bias plots, Passing–Bablock procedure, Cohen’s d, effect sizes, and dependent *t*-tests (*p*-value) (n = 42).

	Bland–Altman Bias ± SD (Lower LOA, Upper LOA)	Passing–Bablok Regression					
		Slope (95% CI)	Proportional Bias	Intercept (95% CI)	Systematic Bias	Cohen *d * (95% CI)	Paired *t*-test (*p*-Value)
LSF	−9.64 ± 34.85 (−77.96, 58.67)	0.9 (0.5, 1.3)	No	8.4 (−53.9, 70.7)	No	0.27 (−0.29, 0.58)	0.38
RSF	−19.22 ± 27.43 (−72.99, 34.55)	0.9 (0.7, 1.2)	No	−6.6 (−52.8, 39.7)	No	0.7 (0.36, 1.03)	<0.001
FLEX	−36.59 ± 45.08 (−124.9, 51.76)	1.1 (0.5, 1.7)	No	−64.5 (−217.3, 88.3)	No	0.81 (0.46, 1.15)	<0.001
EXT	28.44 ± 48.04 (−65.72, 122.6)	1.0 (0.2, 1.8)	No	29.4 (−144.4, 203.1)	No	−0.59 (−0.91, −0.26)	<0.001

EXT = extension; FLEX = flexion; LLF = left-side flexion; RLF = right-side flexion.

## Data Availability

The data that support the findings of this study are available from the corresponding author upon reasonable request.

## Supplementary Materials

The following supporting information can be downloaded at https://www.mdpi.com/article/10.3390/s24155015/s1: Table S1: Inter-session reliability of the isometric neck strength kinematics for the ForceFrame; Figure S1: Comparison between *t*-test for main effect of repetition in flexion [A = Repetition 1 vs. Repetition 2; B = Repetition 1 vs. Repetition 3; C = Repetition 2 vs. Repetition 3].
