# Supplementary material for: Between-Session Reliability of Field-Based Assessments of Isometric Neck Strength
Source: Sensors (Basel). 2024 Aug 2;24(15):5015. doi: 10.3390/s24155015 (PMC11314792; doi:10.3390/s24155015)
Supplement: Supplementary file 1 [file sensors-24-05015-s001.zip › sensors-3067771-supplementary.pdf]

Supplement Table S1 Inter-session reliability of the isometric neck strength kinematics for the ForceFrame

						1–2						2–3						1–3					
			Rep 1	Rep 2	Rep 3		ICC	SEm	MDC	CV			ICC	SEm	MDC	CV			ICC	SEm	MDC	CV	
		Variable	(N)	(N)	(N)	ICC	95 % CI	(N)	(N)	%	95 % CI	ICC	95 % CI	(N)	(N)	%	95 % CI	ICC	95 % CI	(N)	(N)	%	95 % CI
Session 1	EXT	Time to Peak Force (s)	1.47 ± 0.87	1.44 ± 0.81	1.41 ± 0.86	0.74	0.37–0.91	0.42	1.80	48.5	33.2–89.1	0.49	-0.03–0.8	0.59	2.12	66.7	44.9–127.9	0.26	0.14–0.47	0.73	2.37	62.6	42.2–118.7
		Force at 50 ms (N)	90 ± 31	92 ± 40	100 ± 36	0.59	0.11–0.85	22.41	13.12	28.6	20–49.9	0.78	0.44–0.93	17.57	11.62	20.7	14.6–35.4	0.21	0.12–0.39	29.13	14.96	33.5	23.3–59.2
		Force at 100 ms (N)	131 ± 52	133 ± 57	149 ± 54	0.62	0.16–0.86	32.84	15.88	30.3	21.1–53.1	0.84	0.58–0.95	21.87	12.96	18.3	12.9–31.0	0.36	0.19–0.61	42.08	17.98	33.4	23.2–59.1
		Force at 150 ms (N)	161 ± 55	163 ± 54	183 ± 64	0.81	0.50–0.93	23.40	13.41	18.8	13.3–32.0	0.82	0.52–0.94	25.03	13.87	19.8	14.0–33.8	0.58	0.33–0.81	38.69	17.24	26.8	18.8–46.7
		Force at 200 ms (N)	183 ± 57	186 ± 50	200 ± 66	0.82	0.53–0.96	22.25	13.07	16.9	12.0–28.7	0.87	0.65–0.96	20.78	12.64	16.1	11.4–27.2	0.67	0.42–0.86	35.10	16.42	23.4	16.5–40.4
	FLEX	Time to Peak Force (s)	1.53 ± 0.82	1.34 ± 0.78	1.49 ± 0.81	0.54	0.04–0.83	0.45	1.86	54.4	37–101.3	0.83	0.56–0.94	0.32	1.57	35.4	24.5–62.8	0.42	0.23–0.68	0.60	2.15	64.5	43.4–123
		Force at 50 ms (N)	73 ± 18	61 ± 24	66 ± 22	0.01	-0.52–0.51	21.42	12.83	56.3	38.2–105.3	0.04	-0.54–0.49	23.18	13.35	61.9	41.8–117.3	0.30	0.16–0.54	18.18	11.82	29.2	20.4–51.1
		Force at 100 ms (N)	101 ± 29	95 ± 37	88 ± 37	0.29	-0.26–0.70	30.41	15.28	37.7	26.1–67.4	0.57	0.07–0.84	23.68	13.49	35.1	24.4–62.4	0.25	0.14–0.46	28.17	14.71	42.9	29.5–77.7
		Force at 150 ms (N)	127 ± 35	117 ± 50	103 ± 44	0.34	-0.21–0.73	38.50	17.20	42.3	29.1–76.5	0.66	0.22–0.88	27.18	14.45	36.6	25.4–65.3	0.33	0.18–0.58	34.90	16.37	44.2	30.4–80.3
		Force at 200 ms (N)	142 ± 41	130 ± 55	114 ± 46	0.32	-0.23–0.72	44.55	18.50	42.3	29.2–76.6	0.73	0.34–0.90	26.24	14.20	32.3	22.5–57.0	0.40	0.21–0.65	37.16	16.90	41.0	28.3–74.0
	LSF	Time to Peak Force (s)	1.54 ± 0.64	1.84 ± 0.95	1.63 ± 0.80	0.43	-0.11–0.77	0.43	1.83	55.3	37.6–103.2	0.29	-0.27–0.70	0.73	2.37	63.1	42.6–119.9	0.34	0.18–0.59	0.66	2.24	46.9	32.1–85.8
		Force at 50 ms (N)	61 ± 10	65 ± 15	69 ± 16	0.69	0.27–0.89	7.10	7.39	11.8	8.4–19.7	0.86	0.63–0.95	5.67	6.60	9.0	6.5–15.0	0.59	0.34–0.81	8.02	7.85	12.3	8.8–20.6
		Force at 100 ms (N)	82 ± 17	88 ± 32	92 ± 31	0.75	0.38–0.91	11.45	9.38	16.5	11.7–27.9	0.88	0.67–0.96	10.71	9.07	12.4	8.8–20.7	0.58	0.34–0.81	16.38	11.22	16.7	11.9–28.3
		Force at 150 ms (N)	97 ± 23	104 ± 46	112 ± 43	0.78	0.44–0.92	14.83	10.68	18.0	12.7–30.5	0.92	0.76–0.97	12.32	9.73	12.0	8.6–20.0	0.75	0.51–0.90	17.78	11.69	13.9	9.9–23.4
		Force at 200 ms (N)	105 ± 28	115 ± 52	119 ± 47	0.84	0.57–0.94	14.98	10.73	15.8	11.2–26.6	0.93	0.79–0.98	12.75	9.90	11.5	8.2–19.1	0.83	0.62–0.94	16.81	11.37	11.7	8.4–19.5
	RSF	Time to Peak Force (s)	1.64 ± 0.72	1.68 ± 0.91	1.47 ± 0.86	0.14	-0.40–0.61	0.58	2.10	78.4	52.2–154.2	0.64	0.19–0.87	0.52	2.01	47.2	32.2–86.4	0.17	0.11–0.31	0.73	2.37	68.6	46–132
		Force at 50 ms (N)	67 ± 13	65 ± 11	69 ± 15	0.55	0.04–0.83	8.42	8.05	12.8	9.1–21.4	0.91	0.73–0.97	3.91	5.48	6.4	4.6–10.5	0.47	0.26–0.72	8.37	8.02	14.3	10.1–24.0
		Force at 100 ms (N)	83 ± 22	86 ± 20	93 ± 23	0.68	0.26–0.89	13.26	10.09	16.2	10.5–11.5	0.84	0.58–0.95	8.63	8.14	10.5	7.5–17.5	0.70	0.44–0.87	11.49	9.39	11.6	8.3–19.4
		Force at 150 ms (N)	94 ± 30	102 ± 28	111 ± 32	0.75	0.39–0.91	16.07	11.11	16.7	11.8–28.2	0.80	0.49–0.93	13.41	10.15	13.9	9.9–23.3	0.77	0.53–0.91	13.77	10.29	11.6	8.3–19.3
		Force at 200 ms (N)	107 ± 34	112 ± 33	125 ± 36	0.84	0.58–0.95	14.96	10.72	13.6	9.7–22.9	0.85	0.61–0.95	13.33	10.12	12.7	9.0–21.2	0.80	0.57–0.93	14.70	10.63	10.9	7.8–18.2
Session 2	EXT	Time to Peak Force (s)	1.53 ± 0.48	1.23 ± 0.59	1.45 ± 0.73	0.04	-0.48–0.54	0.57	2.10	57.6	39–108	0.47	0.38–0.84	0.48	1.92	54.1	36.8–100.7	0.72	0.38–0.84	0.29	1.49	36.8	25.5–65.6
		Force at 50 ms (N)	80 ± 29	79 ± 29	78 ± 25	0.72	0.32–0.90	15.86	11.04	21.3	15–36.5	0.72	0.33–0.9	14.09	10.40	22.2	15.6–38.1	0.55	0.31–0.78	19.00	12.08	21.6	15.3–37.1
		Force at 100 ms (N)	118 ± 40	121 ± 46	125 ± 48	0.77	0.43–0.92	21.17	12.75	22.3	15.7–38.4	0.72	0.33–0.90	24.50	13.72	27.7	19.4–48.2	0.54	0.30–0.78	28.83	14.88	28.0	19.6–48.8
		Force at 150 ms (N)	150 ± 50	156 ± 58	164 ± 68	0.75	0.39–0.91	25.34	13.95	22.1	15.6–37.9	0.72	0.34–0.90	32.97	15.92	26.0	18.2–45.1	0.45	0.24–0.71	39.56	17.43	32.4	22.6–57.2

	Force at 200 ms (N)	174 ± 57	178 ± 66	184 ± 72	0.78	0.45–0.92	25.57	14.02	18.9	13.4–32.2	0.77	0.43–0.92	32.47	15.79	20.4	14.4–34.9	0.44	0.23–0.69	45.24	18.64	29.3	20.4–51.2
FLEX	Time to Peak Force (s)	1.38 ± 0.69	1.44 ± 0.75	1.59 ± 0.60	0.34	-0.22–0.72	0.56	2.07	64.2	43.3–122.3	0.26	-0.29–0.69	0.58	2.11	62.9	42.4–119.5	0.35	0.19–0.60	0.57	2.10	35.9	24.9–63.9
	Force at 50 ms (N)	70 ± 17	67 ± 16	72 ± 17	0.67	0.25–0.88	8.79	8.22	15.9	11.3–26.9	0.41	-0.13–0.76	12.40	9.76	20.9	14.7–35.8	0.63	0.38–0.84	9.68	8.63	12.4	8.8 -20.7
	Force at 100 ms (N)	100 ± 29	99 ± 30	102 ± 26	0.90	0.72–0.97	8.23	7.95	12.3	8.8–20.6	0.49	-0.04–0.80	19.61	12.27	25.5	17.9- 44.3	0.34	0.18–0.58	22.67	13.20	21.3	15.0–36.4
	Force at 150 ms (N)	125 ± 39	122 ± 40	129 ± 33	0.92	0.77–0.97	9.65	8.61	12.2	8.7–20.3	0.67	0.23–0.88	20.65	12.60	21.1	14.9–36.1	0.24	0.14–0.44	31.79	15.63	23.8	16.7–41.0
	Force at 200 ms (N)	141 ± 45	140 ± 47	142 ± 33	0.90	0.72–0.97	12.23	9.69	13.4	9.6–22.5	0.74	0.36–0.91	20.25	12.47	17.4	12.3–29.5	0.01	0.05 - -0.25	42.14	17.99	26.3	18.4–45.6
LSF	Time to Peak Force (s)	1.54 ± 0.68	1.49 ± 0.47	1.42 ± 0.89	0.28	-0.28–0.69	0.55	2.06	47.5	32.6–87.1	0.31	-0.24–0.71	0.58	2.11	61.5	41.5–116.4	0.46	0.25–0.71	0.42	1.80	59.8	40.4–112.7
	Force at 50 ms (N)	62 ± 14	59 ± 14	58 ± 11	0.88	0.66–0.96	4.46	5.85	8.5	6.1–14.0	0.79	0.46–0.93	5.66	6.60	10.3	7.4–17.2	0.50	0.28–0.75	9.83	8.69	10.9	7.8–18.2
	Force at 100 ms (N)	83 ± 24	76 ± 29	79 ± 25	0.85	0.59–0.95	9.22	8.42	13.9	9.9–23.3	0.95	0.85–0.98	5.99	6.78	8.2	5.9–13.6	0.70	0.44–0.88	14.43	10.53	14.0	10.0–23.5
	Force at 150 ms (N)	97 ± 32	89 ± 40	97 ± 36	0.81	0.50–0.93	14.52	10.56	18.3	13.0–31.1	0.97	0.90–0.99	6.56	7.10	7.7	5.6–12.8	0.72	0.47–0.89	19.01	12.09	16.0	11.4–27.1
	Force at 200 ms (N)	107 ± 37	97 ± 46	107 ± 45	0.84	0.57–0.94	15.36	10.86	17.1	12.1–28.9	0.96	0.88–0.99	8.96	8.30	8.8	6.3–14.5	0.78	0.54–0.91	19.37	12.20	15.5	11.0–26.1
RSF	Time to Peak Force (s)	1.57 ± 0.94	1.55 ± 0.68	1.47 ± 0.62	0.59	0.11–0.58	0.50	1.95	56.4	38.3–105.6	0.84	0.58–0.95	0.26	1.40	29.1	20.4–50.9	0.22	0.13–0.41	0.71	2.34	54.5	37.1 -101.6
	Force at 50 ms (N)	66 ± 10	62 ± 14	73 ± 15	0.67	0.24–0.88	6.10	6.85	12.6	9.0–21.0	0.76	0.41–0.92	7.45	7.57	12.2	8.7–20.3	0.29	0.16–0.52	10.48	8.98	17.9	12.7–30.3
	Force at 100 ms (N)	89 ± 19	85 ± 28	99 ± 30	0.73	0.35–0.91	10.10	8.81	17.3	12.2–29.2	0.70	0.30–0.89	16.05	11.11	21.5	15.1–36.8	0.59	0.34–0.81	15.28	10.84	19.4	13.7–33.0
	Force at 150 ms (N)	110 ± 31	101 ± 39	117 ± 39	0.85	0.59–0.95	11.45	9.38	16.2	11.5–27.3	0.73	0.36–0.91	20.11	12.43	23.6	16.6–40.6	0.72	0.47–0.89	18.40	11.89	16.9	12.0–28.7
	Force at 200 ms (N)	122 ± 36	114 ± 47	126 ± 44	0.87	0.64–0.96	12.24	9.70	15.9	11.3–26.9	0.75	0.38–0.91	22.45	13.13	24.5	17.2–42.3	0.76	0.52–0.91	20.14	12.44	15.7	11.2–26.5

ICC = intraclass correlation coefficient; 95% confidence interval for the $ICC_{(3,1)}$ single measure; SE_m = standard error of measurement; MDC = minimal detectable change; Ext = extension; Flex = flexion; LSF = left-side flexion; RSF = right-side flexion.

.

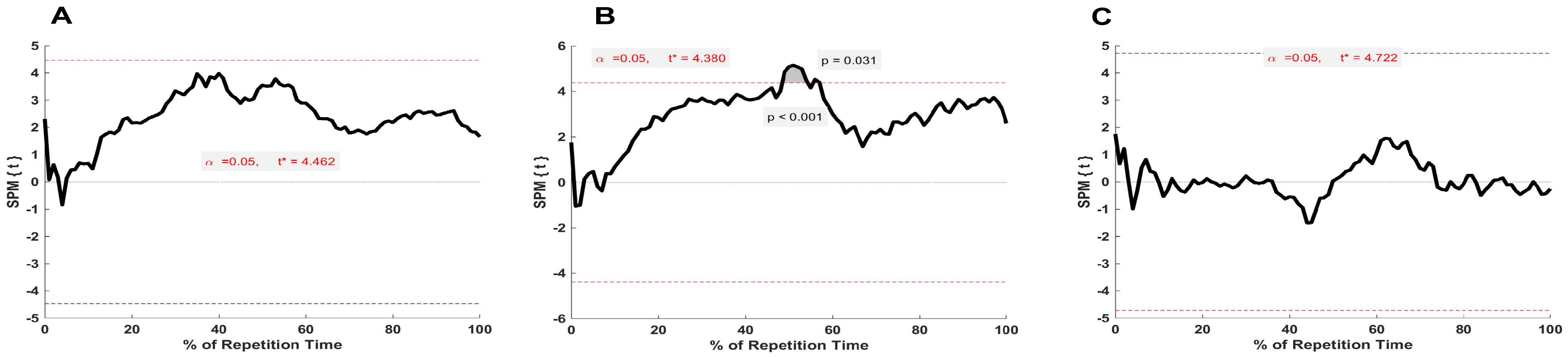

Supplement Figure S1. Comparison between t-test for main effect of repetition in flexion [A = Repetition 1 vs. Repetition 2; B = Repetition 1 vs. Repetition 3; C = Repetition 2 vs Repetition 3].
